# Cardiopulmonary Exercise Testing and HFpEF: Diagnostic and Therapeutic Perspectives

**DOI:** 10.3390/healthcare13233098

**Published:** 2025-11-27

**Authors:** Francesco Di Spigno, Valeria Dall’Ospedale, Luigi Gerra, Federico Breviario, Andrea Tedeschi, Giancarlo Trimarchi, Daniela Aschieri

**Affiliations:** 1 Cardiology Unit, Guglielmo da Saliceto Hospital, 29121 Piacenza, Italy; valeriadallospedale@gmail.com (V.D.); l.gerra@ausl.pc.it (L.G.); f.breviario@ausl.pc.it (F.B.); a.tedeschi@ausl.pc.it (A.T.); 2 Interdisciplinary Center for Health Sciences, Scuola Superiore Sant’Anna, 56100 Pisa, Italy; giancarlo.trimarchi18@gmail.com; 3 Heart Centre, Cardiology Unit, Fondazione Gabriele Monasterio, 54100 Massa, Italy

**Keywords:** heart failure with preserved ejection fraction, HFpEF, cardiopulmonary exercise testing, CPET, peak oxygen uptake, oxygen pulse, ventilatory efficiency, VE/VCO_2_ slope, prognostic assessment, exercise intolerance

## Abstract

Background: Heart failure with preserved ejection fraction (HFpEF) is a complex and heterogeneous clinical syndrome that represents an increasing global health challenge due to its high rates of morbidity, hospitalization, and mortality. In this context, cardiopulmonary exercise testing (CPET) has emerged as a valuable diagnostic modality, particularly in patients presenting with unexplained exertional dyspnea and inconclusive resting imaging results. Objectives and Clinical Implications: This narrative review examines current evidence on the clinical relevance and prognostic value of CPET parameters in HFpEF. Peak VO_2_, a marker of aerobic capacity and cardiovascular fitness, has established prognostic value in heart failure with reduced ejection fraction (HFrEF). However, its prognostic significance in HFpEF remains less well defined. Oxygen pulse has emerged as another important measure for evaluating functional capacity and predicting response to exercise-based interventions, offering potential prognostic insight into adverse outcomes in HFpEF. Finally, the VE/VCO_2_ slope, an index of ventilatory efficiency during exercise, also holds clinical relevance in HFpEF, particularly with concomitant pulmonary hypertension, though evidence remains heterogeneous. Conclusions: CPET-derived variables are valuable parameters for HFpEF assessment. Their systematic integration into clinical evaluation of HFpEF patients could guide individualized management strategies and inform clinicians for improving outcomes in this challenging condition.

## 1. Introduction

Heart failure with preserved ejection fraction (HFpEF) is a complex and heterogeneous syndrome defined by typical heart failure symptoms and signs in the context of a left ventricular ejection fraction (LVEF) ≥ 50%. According to the universal definition of heart failure, it involves structural and/or functional cardiac abnormalities leading to systemic or pulmonary congestion, supported by elevated natriuretic peptides [1,2].

HFpEF represents a growing global health burden due to its association with high morbidity, hospitalizations, and mortality [2]. Reliable epidemiological data on the incidence, prevalence, and prognosis of chronic heart failure in the general population remain limited. Approximately half of all heart failure cases are classified as HFpEF, with prevalence increasing due to aging populations [3].

In the EPICA cohort, about 40% of HF cases were attributed to preserved systolic function, 30% to systolic dysfunction, 16% to valvular heart disease, 11% to right heart failure, 2% to multifactorial causes, and 1% to pericardial disease [4].

In addition to its higher prevalence among the elderly, data from a southwestern European community show that at any given age, HFpEF is more common in women than men [3]. Similarly, studies from the United States have consistently demonstrated that women have slightly higher age-adjusted incidence rates of HFpEF than men [5].

Patients with HFpEF typically exhibit a higher degree of multimorbidity than those with HFrEF, with approximately 50% affected by five or more major comorbidities. The most common risk factors include hypertension, obesity, and coronary artery disease, with atrial fibrillation also frequently present. Notably, diabetes does not appear to be an independent predictor of HFpEF once adjusted for other variables [3].

Mortality estimates for HFpEF vary widely depending on the study design and patient characteristics, such as inpatient versus outpatient status. In-hospital mortality ranges from 2.4% to 4.9%, while 30-day and 60–90-day mortality rates are around 5% and 9.5%, respectively. One-year mortality ranges between 20% and 29%, with higher rates observed in patients previously hospitalized. By five years, at least 50% of patients with HFpEF have died, with mortality estimates between 53% and 74%. Despite these figures, large randomized clinical trials report lower annualized mortality rates of 4–5 per 100 person-years. In all settings, mortality in HFpEF remains higher than in the general population [3].

The most widely implicated mechanism in HFpEF is diastolic dysfunction, which involves impairments in both active relaxation and passive stiffness of the myocardium, ultimately leading to elevated filling pressures [6]. Myocardial remodeling and associated diastolic dysfunction may be driven by a systemic pro-inflammatory state triggered by common HFpEF comorbidities, especially obesity and metabolic syndrome [7]. More recently, it has been recognized that this pro-inflammatory response is also partially mediated by hemodynamic alterations, such as arterial hypertension or aortic stenosis [8]. The increased expression of adhesion molecules and E-selectin on the vascular endothelium, as part of this inflammatory state, stimulates TGF-beta production, which activates fibroblasts to increase collagen synthesis. Additionally, mechanical stress may lead to a phenotypic change in resident fibroblasts, promoting basal lamina remodeling.

However, extracellular fibrosis alone may not be the predominant mechanism driving myocardial stiffness. In a large study based on endomyocardial biopsies from HFpEF patients, fibrosis was found to be absent or mild in 73% of cases [9]. Part of myocardial stiffness may be attributed to excessive production of reactive oxygen species (ROS) by endothelial cells of the coronary microcirculation, mediated by multiple cytokines, including GDF-15 and TNF-alpha.

Myocardial stiffness causes elevated filling pressures which result in pulmonary hypertension and lead to right ventricular remodeling and dysfunction, ultimately contributing to systemic congestion. Right ventricular dysfunction is a key marker of HFpEF progression and is associated with poor prognosis [10]. Regarding exercise intolerance, patients with diastolic dysfunction—even those with little or no elevation in left ventricular filling pressure at rest—experience a substantial increase in left ventricular diastolic pressures and pulmonary venous pressures during exertion, significantly limiting their exercise capacity.

Tachycardia during exercise shortens diastole, reducing the available time for left ventricular filling [11].

Moreover, a large proportion of HFpEF patients exhibit chronotropic incompetence, which limits the heart rate response to exercise and restricts increases in cardiac output. Besides reduced oxygen delivery, peripheral tissues also demonstrate diminished oxygen extraction, likely due to systemic inflammation and chronic oxidative stress, which also increase vascular stiffness [12].

Skeletal and respiratory muscles are also affected: mitochondrial dysfunction reduces ATP production, calcium homeostasis is impaired, and oxidative stress is increased [13].

The level of exercise intolerance in patients with HFpEF is comparable to those with heart failure with reduced ejection fraction (HFrEF), as both conditions involve disruptions in the oxygen uptake (VO_2_) process and abnormal physiological responses across several organ systems [14]. Accurate identification and objective assessment of both cardiac and extracardiac factors associated with exercise limitation and VO_2_ reduction are essential for a tailored diagnosis and therapy. In this context, Cardiopulmonary Exercise Testing (CPET) with gas exchange analysis is considered the gold standard for non-invasive assessment of functional capacity and provides valuable insight into how lung mechanics and cardiopulmonary interactions contribute to muscle weakness. However, the role of CPET in HFpEF management is still unclear [15]. Given these diagnostic challenges, this review aims to synthesize evidence on key CPET-derived variables for HFpEF assessment.

## 2. CPET and HFpEF

Cardiopulmonary exercise testing (CPET) represents a valuable and objective method for assessing functional capacity and has demonstrated prognostic relevance in predicting hospitalizations for HF, adverse clinical outcomes, and mortality in patients with HF [16,17,18,19]. In the context of HFpEF, CPET offers insight into the mechanisms underlying exercise intolerance, one of the cardinal clinical features of the syndrome. Specifically, reduced exercise capacity is commonly defined by a peak oxygen consumption (VO_2_ max) ≤ 20 mL/kg/min, while ventilatory inefficiency is often represented by a VE/VCO_2_ slope ≥ 30 [17].

CPET enables the quantification of exercise limitation and may assist in differentiating cardiac from non-cardiac (e.g., pulmonary or peripheral) etiologies of exertional dyspnea. However, its prognostic value and its ability to discriminate between HFpEF and non-cardiac causes of exercise intolerance remains uncertain. Notably, a peak VO_2_ value < 75% of the predicted reference has been employed in several HFpEF trials to identify patients with clinically significant exercise impairment [20,21]. The underlying mechanisms responsible for reduced oxygen uptake in HFpEF are multifactorial and involve central, peripheral, and microvascular dysfunction [12,13].

Despite the growing interest in CPET, its routine incorporation into the diagnostic workup for HFpEF remains uncommon, and its utility in this specific patient population is not as well established as in HFrEF [22,23,24]. To date, there is no consensus regarding the optimal CPET-derived cut-off values for diagnosing HFpEF, nor is there clarity on which parameters possess the strongest prognostic significance in terms of hospitalization risk or mortality. As a result, current guidelines provide limited and non-specific recommendations for the role of CPET in HFpEF [25,26].

The variability in findings across studies is partly attributable to the heterogeneity of the HFpEF population and the lack of uniform diagnostic criteria. Many earlier studies were retrospective and based on inconsistent definitions of HFpEF [27,28]. Only two recent investigations [29,30] have applied the Heart Failure Association’s (HFA) consensus definition using the HFA-PEFF diagnostic algorithm [26], while prior studies defined HFpEF solely on sign and symptoms of HF diastolic dysfunction and a left ventricular ejection fraction (LVEF) > 50% [31]

In this narrative review, we focus on three core CPET-derived variables, peak oxygen uptake (peak VO_2_), oxygen pulse (VO_2_/heart rate), and ventilatory efficiency (VE/VCO_2_ slope)—which have emerged as the most promising parameters for both the characterization of HFpEF and the assessment of its prognostic trajectory. Table 1 summarizes the main studies on CPET in HFpEF patients and its prognostic value.

**Figure 1:**
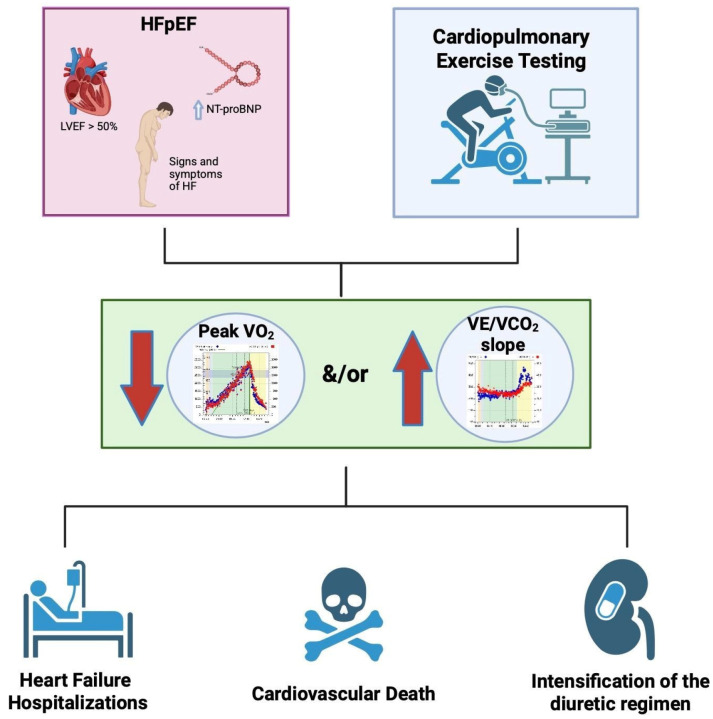
Schematic flow diagram summarizing the interpretation of key CPET parameters in patients with HFpEF and their prognostic implications.

To provide a concise overview of CPET interpretation in HFpEF, we present a flow diagram in Figure 1.

Table 1 CPET studies in HFpEF. Parameters considered, cut-off values and their prognostic role. ACS = Acute Coronary Syndrome; AHF = Acute Heart Failure; AF = Atrial Fibrillation; AV = Atrio-Ventricular; CKD = Chronic Kidney Disease; CM = cardiomyopathy; CO = Cardiac Output; COPD = Chronic Obstructive Pulmonary Disease; CTx = Cardiac Transplant; CV = Cardiovascular; DHF = Diastolic Heart Failure; EOV = Exertional Oscillatory Ventilation; HCM = Hypertrophic Cardiomyopathy; HF = Heart Failure; HFmrEF = Heart Failure with mildly reduced Ejection Fraction; HFpEF = Heart Failure with preserved Ejection Fraction; HFrEF = Heart Failure with reduced Ejection Fraction; HFA-PEFF = Heart Failure Association Pre-test assessment, Echocardiography & natriuretic peptides, Functional testing, Final aetiology (diagnostic algorithm for HFpEF); HR = Heart Rate; IDI = Integrated Discrimination Improvement; IRR = Incidence Rate Ratio; IV = Intravenous; LVAD = Left Ventricular Assist Device; LVDD = Left Ventricular Diastolic Dysfunction; LVEF = Left Ventricular Ejection Fraction; NRI = Net Reclassification Improvement; NYHA = New York Heart Association; PCWP = Pulmonary Capillary Wedge Pressure; ppMVO_2_ = percentage of predicted peak VO_2_; %PredO_2_P = Percent Predicted Oxygen Pulse; PVR = Pulmonary Vascular Resistance; SHF = Systolic Heart Failure; VE/VCO_2_ = Ventilatory Equivalent for Carbon Dioxide; VO_2_ = Oxygen Consumption

## 3. Maximal O_2_ Uptake-VO_2_ Peak

Peak VO_2_ represents a fundamental parameter in CPET, reflecting an individual’s aerobic capacity and overall cardiovascular fitness. Its prognostic utility is well established in patients with HFrEF, where it also serves as a key determinant in guiding advanced therapeutic decisions, such as timing for cardiac transplant (CTx) [25]. When assessed invasively, peakVO_2_ is considered the gold standard for evaluating functional capacity also in HFpEF [38,39]. However, the body of evidence supporting its prognostic significance when measured during CPET in HFpEF remains relatively limited (Table 1).

In a prospective study involving 173 patients with HFpEF who underwent CPET and were followed for a median of 5.2 years, Shafiq et al. demonstrated that lower peak VO_2_ values were associated with adverse clinical outcomes, with patients experiencing events exhibiting significantly reduced values compared to event-free individuals (14.0 vs. 17.9 mL/min/kg) [27]. Similarly, Zern et al. reported that HFpEF patients had lower peak VO_2_ compared to hypertensive control subjects (15.1 vs. 20.7 mL/min/kg) [40]. Furthermore, among HFpEF patients, those with chronotropic incompetence showed significantly reduced peak VO_2_ compared to those without this condition (9.6 vs. 12.5 mL/min/kg), highlighting the influence of chronotropic response on exercise performance and O_2_ extraction in HFpEF [40].

Beyond absolute peak VO_2_, increasing attention has been given to normalized indices such as percent predicted peak VO_2_ (ppMVO_2_). In a study conducted by Palau et al. involving 74 stable and symptomatic patients with HFpEF, a 10% reduction in ppMVO_2_ was independently associated with a 32% increased risk of recurrent hospitalization over a median follow-up period of 276 days [34]. This association remained linear and statistically significant, underscoring the value of ppMVO_2_ as a prognostic marker for adverse outcomes in HFpEF. These findings are consistent with those of Shafiq et al., who reported that ppMVO_2_ served as a more powerful prognostic indicator than absolute peak VO_2_, thereby supporting the clinical relevance of adjusted CPET metrics [27].

In a recent study with HFpEF patients diagnosed with HFA-PEFF score criteria, Naito et al. [29] demonstrated that low peak VO_2_ was strongly associated with increased risk of adverse outcomes (HR 5.05, 95% CI 2.65–9.62).

Nevertheless, the prognostic utility of peak VO_2_ in HFpEF remains a topic of ongoing debate. In another recent study involving 99 HFpEF patients diagnosed according to the HFA-PEFF score criteria [30], individuals with a mean peak VO_2_ < 14 mL/min/kg exhibited worse baseline clinical profiles, including higher body mass index (BMI), increased prevalence of diabetes, greater symptom burden (NYHA class III), elevated NT-proBNP levels, reduced estimated glomerular filtration rate (eGFR), increased E/e′ ratio, and lower resting heart rate. Despite these associations, after a follow-up period exceeding two years, a mean peak VO_2_ < 14 mL/min/kg did not demonstrate a statistically significant association with the composite outcome of HF hospitalization or CV death (HR: 1.34; 95% CI: 0.60–2.99). Similarly, Guazzi et al. [31] previously reported no significant correlation between peak VO_2_ and adverse outcomes, namely all-cause mortality and hospitalizations, in patients with HFpEF.

These mixed findings reflect the complexity of HFpEF, indeed, reduced peak VO_2_ is highly sensitive but not specific, and it differentiates HFpEF from non-cardiac dyspnea reliably only when the values are markedly high or low [41]. This highlights the need for further prospective studies to clarify the independent prognostic role of peak VO_2_ and its derived indices in this heterogeneous population.

## 4. O_2_ Pulse-Peak VO_2_/HR

In individuals with HFpEF, O_2_ pulse has emerged as a relevant parameter for assessing functional capacity and predicting responsiveness to exercise-based interventions. Defined as the ratio between peak oxygen uptake (VO_2_) and heart rate, O_2_ pulse serves as an indirect measure of stroke volume and reflects changes in left ventricular performance during physical exertion [42].

Li et al. [32] demonstrated that, in a cohort of 145 patients with LVEF ≥ 50% and resting supine PCWP ≥ 15 mmHg and/or exercise PCWP/CO ≥ 2.0 mm Hg/L/min, percent predicted peak O_2_ pulse (%PredO_2_P) < 85% was an independent prognostic marker for all-cause death and those with higher %PredO_2_P exhibited longer survival.

In this context, %PredO_2_P offers a valuable, non-invasive metric that correlates with key hemodynamic variables, including stroke volume, oxygen utilization efficiency, and peak VO_2_. Elevated %PredO_2_P values are associated with enhanced exercise performance and superior cardiovascular functional reserve in HFpEF populations.

Recent evidence has further supported the concurrent validity of stroke volume estimation via various modalities—including SV_ACET, SV_ECHO, and O_2_ pulse—underscoring their clinical utility in approximating exercise-induced stroke volume in this patient cohort [43]. Notably, changes in peak O_2_ pulse have been shown to explain approximately 72% of the variance in peak VO_2_ improvements observed between exercise-trained patients and control groups, thereby highlighting its potential as a surrogate marker for estimate exercise responsiveness [32].

The administration of beta-blockers in HFpEF has been linked to blunted increases in O_2_ pulse during exertion, potentially impairing exercise tolerance [44]. As such, quantification of O_2_ pulse dynamics may aid in stratifying patients and optimizing therapeutic strategies [44]. Furthermore, an emerging body of literature suggests that peak O_2_ pulse (VO_2_/HR), by reflecting stroke volume at maximal exertion, may also be associated with neurocognitive outcomes in HFpEF, consistent with the vascular cascade hypothesis [45].

O_2_ pulse, as a determinant of the Fick equation, encapsulates both central and peripheral components of oxygen delivery. Compared to VO_2_ or ventilatory efficiency, it may offer enhanced prognostic utility in predicting adverse clinical outcomes in patients with HFpEF [46].

## 5. Ventilatory Efficiency-VE/VCO_2_ Slope

The VE/VCO_2_ slope, or the ventilatory equivalent for carbon dioxide, is a key CPET parameter that reflects ventilatory efficiency during exercise and holds significant clinical and prognostic relevance in patients with HFpEF.

Patients with HFpEF typically exhibit an elevated VE/VCO_2_ slope compared to healthy individuals, indicative of ventilatory inefficiency during physical exertion. While this phenomenon is also observed in HFrEF, the underlying pathophysiological mechanisms differ. In HFpEF, the increased VE/VCO_2_ slope is predominantly attributed to a higher physiological dead space to tidal volume ratio (VD/VT) and altered arterial carbon dioxide tension (PaCO_2_), often due to pulmonary vascular dysfunction and comorbid pulmonary hypertension [47].

The VE/VCO_2_ slope may be considered a diagnostic element for HFpEF. In hypertensive patients with exertional symptoms, a cutoff value of 32.95 yielded 100% sensitivity and 90% specificity for identifying HFpEF [48], indicating its potential in the early detection of subclinical disease.

From a prognostic standpoint, the VE/VCO_2_ slope has demonstrated predictive utility in selected HFpEF cohorts, particularly those with concurrent pulmonary hypertension. Threshold values above 30–33 have been associated with an increased risk of adverse events. In two recent studies applying the HFA-PEFF score to diagnose HFpEF, Rozados da Conceicao et al. [30] and Naito et al. [29] demonstrated that VE/VCO_2_ slope was significantly associated with HF hospitalization, worsening HF, CV, and all-cause death when above 34 (HR 2.69, 95% CI 1.00–7.2) and 45 (HR 4.59, 95% CI 2.24–9.40), respectively, in patients with HFpEF.

Similar findings have been reported by other studies [28,33] where HFpEF was diagnosed in the presence of signs and symptoms of congestive HF and LVEF ≥ 50%. In these cases, VE/VCO_2_ slope independently predicted HF hospitalization and all-cause death.

In other cohorts, VE/VCO_2_ slope demonstrated even greater prognostic value compared with peakVO_2_. In 88 patients with HFpEF and signs of pulmonary hypertension, increased VE/VCO_2_ slope (but not peak VO_2_) was independently associated with all-cause death [35]. Moreover, Yan et al. [36], in a cohort of 224 patients, showed that a VE/VCO_2_ slope ≥ 34.7, on multivariate analysis (but not peakVO_2_) was independently associated with higher risk of CV death (HR 1.02, 95% CI 1.01–1.04) and all-cause death (HR 1.03, 95% CI 1.01–1.05).

Despite the evidence presented, the prognostic role of the VE/VCO_2_ slope in HFpEF has not always been fully established. For example, in a study of 173 patients, the VE/VCO_2_ slope was not significantly associated with the outcome of all-cause death or heart transplantation [27].

In summary, the VE/VCO_2_ slope is an important and easily obtainable CPET parameter that provides insight into ventilatory efficiency, exercise limitation mechanisms, and risk stratification in HFpEF. Nevertheless, further prospective studies are warranted to standardize its application and refine its interpretive value across different HF populations. Finally, the VE/VCO_2_ slope appears to have a recognized prognostic significance in HFpEF, comparable to that observed in HFrEF, although its clinical interpretation should probably consider the influence of age and sex differences.

## 6. Exercise Oscillatory Ventilation (EOV)

Exercise oscillatory ventilation (EOV) is a well-recognized abnormal breathing pattern that can be observed in HF, especially in HFrEF, where it is associated with a poor prognosis [49]. Pathophysiologically, EOV reflects unstable ventilatory control—heightened chemosensitivity, prolonged circulatory time, pulmonary congestion, similar to Cheyne–Stokes respiration. EOV may reflect autonomic imbalance and persistently elevated sympathetic drive [49]. Patients exhibiting EOV typically have clinical features and exercise ventilatory responses consistent with more advanced HF, even when left ventricular function is comparable to that of patients without EOV [50].

Multiple definitions of EOV exist, most relying on arbitrary cut-offs for the amplitude and duration of visually apparent oscillations. In general, EOV is defined by cyclic fluctuations in minute ventilation (VE) that occur with notable regularity and persist for a significant portion of the exercise phase. Kremser et al. conducted the first study of EOV in HF [51]. The authors evaluated 31 patients with chronic congestive HF and identified EOV in 6 cases, defined as oscillations in VE exceeding 15% of the mean resting value, present at rest and persisting for more than two-thirds (>66%) of the CPET duration. This small subgroup exhibited a significantly lower VO_2_peak than those without EOV, suggesting that EOV marked more advanced HF, probably reflecting inadequate oxygen delivery from reduced cardiac output. The American Heart Association (AHA) consensus statement defined EOV as an oscillatory ventilatory pattern that persists for at least 60% of the exercise test, with an amplitude ≥ 15% of the average resting value [52]. Because automated measurement methods are lacking, the presence of EOV during CPET is usually assessed visually, which may have led to variability in definitions and identification; adherence to standardized thresholds can reduce inter-observer variability and improve reproducibility of CPET interpretations [52].

From a physiological point of view, EOV reflects instability of ventilatory control mechanisms. Contributing factors include altered chemoreceptor sensitivity, delayed circulation time between lungs and peripheral receptors, pulmonary congestion with stimulation of J-receptors, and increased physiological dead space [53]. In HFrEF, EOV has been consistently associated with greater disease severity, ventilatory inefficiency (elevated VE/VCO_2_ slope), reduced exercise tolerance, and adverse outcomes, including mortality and hospitalization [53,54].

Conversely, the role of EOV in heart failure with preserved ejection fraction (HFpEF) is less certain. In a 2008 study of 556 HF patients (151 HFpEF and 405 HFrEF), Guazzi et al. reported similar EOV prevalence in HFpEF and HFrEF (31% vs. 35%). On univariable Cox analysis, VO_2_peak, the VE/VCO_2_ slope, and EOV each predicted cardiac events in both groups, particularly EOV (*p* < 0.001 in each group). In multivariable models, EOV remained an independent prognostic marker in HFrEF and was the strongest predictor of cardiac events in HFpEF [37].

Findings from Shafiq et al. [27] differed. This retrospective analysis included patients with HFpEF (ejection fraction ≥ 50%) who underwent CPET between 1997 and 2010. Selected variables included VO_2_peak, percent-predicted VO_2_peak (ppMVO_2_), the VE/VCO_2_ slope, and EOV. Separate Cox regression analyses assessed associations with a composite outcome of all-cause mortality or heart transplantation (HTx) [27]. In this cohort, variables known to be prognostic in HFrEF (VO_2_peak and ppMVO_2_) were also significantly associated with the composite outcome in HFpEF. Among the CPET variables, ppMVO_2_ was the best prognostic indicator, conferring a 30% lower risk per 10% increase in ppMVO_2_, whereas EOV (*p* = 0.70) showed no significant association with the composite endpoint [27].

Overall, the limited HFpEF evidence indicate that EOV should be considered an adjunctive marker—interpreted alongside VO_2_peak/ppMVO_2_ and the VE/VCO_2_ slope rather than a stand-alone discriminator of risk. More HFpEF-specific studies using standardized EOV definitions and preferably automated detection are needed to clarify its incremental value beyond clinical, echocardiographic, and CPET variables.

## 7. Peripheral Oxygen Extraction

In patients with HFpEF, impaired peripheral oxygen extraction during exercise has emerged as a critical determinant of reduced exercise capacity [12]. Insights from CPET combined with invasive hemodynamic measurements have underscored the relevance of this peripheral limitation. Specifically, peak arteriovenous oxygen content difference [C(a–v)O_2_] has been identified as a major determinant of aerobic performance in HFpEF [12].

Compared to individuals with HFrEF and healthy controls, HFpEF patients frequently exhibit a reduced peak exercise C(a–v)O_2_, indicating diminished oxygen extraction by skeletal muscle during exertion. This limitation persists despite preserved or even augmented cardiac output responses, suggesting that central hemodynamics may not be the primary constraint in these individuals [12].

It is estimated that 40% to 75% of HFpEF patients exhibit impaired peripheral oxygen extraction as the predominant mechanism underlying exercise intolerance. This dysfunction has been attributed to several factors, including skeletal muscle mitochondrial abnormalities, microvascular rarefaction, and reduced oxygen diffusive conductance [12].

Studies employing isolated muscle exercise protocols—such as single-leg knee extension, which minimize central cardiac limitations—have shown that HFpEF patients classified as peripherally limited demonstrate reduced limb blood flow, lower arteriovenous oxygen gradients, and impaired oxidative metabolism, as assessed by phosphorus magnetic resonance spectroscopy [55].

Moreover, impaired peripheral oxygen utilization correlates with delayed oxygen uptake kinetics during submaximal exercise and an exaggerated hypertensive response, both of which contribute to exertional dyspnea and reduced functional capacity in this population [56].

## 8. Clinical Implications

The diagnosis of HFpEF remains a significant clinical challenge due to its complex and heterogeneous pathophysiology, as well as the absence of a definitive diagnostic test. Patients frequently present with nonspecific symptoms such as exertional dyspnea, fatigue, and diminished exercise tolerance, clinical features that overlap with a variety of non-cardiac conditions, particularly among elderly individuals with multiple comorbidities. Accordingly, a systematic, multimodal diagnostic approach is recommended, integrating clinical evaluation with biomarker analysis, imaging modalities, and functional assessments.

Natriuretic peptides, particularly B-type natriuretic peptide (BNP) and N-terminal proBNP (NT-proBNP), serve as useful initial biomarkers; elevated levels support the diagnosis of HFpEF in the appropriate clinical setting. However, their interpretation can be confounded by factors such as obesity and atrial fibrillation, necessitating additional diagnostic modalities [57]. Resting transthoracic echocardiography remains central to the structural and functional evaluation of suspected HFpEF, allowing assessment of diastolic dysfunction, left ventricular hypertrophy, left atrial enlargement, and elevated filling pressures [57,58].

In cases where initial evaluations yield inconclusive findings, particularly in patients with unexplained exertional symptoms, CPET has emerged as a valuable diagnostic adjunct [59]. CPET represents a valuable and objective diagnostic modality that should be regarded as an integral component of the diagnostic and therapeutic pathway for patients with HFpEF.

The principal utility of CPET lies in its unique capacity to elucidate the underlying pathophysiology of exercise limitation. By simultaneously measuring ventilatory gas exchange, cardiovascular function, and metabolic parameters, CPET enables clinicians to discriminate between cardiac and non-cardiac (e.g., pulmonary, peripheral vascular, or skeletal muscle) etiologies of dyspnoea and fatigue. Specifically, the analysis of key variables such as peak oxygen consumption (peak VO_2_), the ventilatory equivalent for carbon dioxide (VE/VCO_2_ slope), and end-tidal carbon dioxide pressure (PetCO_2_) and VO_2_/heart rate provides a window into the efficacy of oxygen delivery and utilization. These parameters may play a role in the early identification of subclinical functional limitation, even before overt signs of congestion appear and can reveal early abnormalities in cardiovascular reserve and ventilatory efficiency that are not necessarily correlated with NYHA class or resting echocardiographic parameters. Peak VO_2_ is a well-established indicator of aerobic capacity and cardiovascular performance, with validated prognostic relevance in HFrEF, where it also guides decisions regarding advanced therapies such as heart transplantation. In HFpEF, however, the prognostic utility of peak VO_2_ is less clearly defined and warrants further investigation. Oxygen pulse has been recognized as a meaningful metric for assessing functional capacity and forecasting responsiveness to exercise interventions. It may also provide incremental prognostic value regarding adverse clinical outcomes in HFpEF. The VE/VCO_2_ slope, an indicator of ventilatory efficiency during exertion, has demonstrated clinical relevance in HFpEF, especially among patients with coexistent pulmonary hypertension. Nevertheless, its prognostic significance remains inconsistent across studies and continues to evolve.

To improve diagnostic precision in HFpEF, structured tools such as the H_2_FPEF score and the HFA-PEFF algorithm have been developed. The H_2_FPEF score offers a practical approach to estimating the likelihood of HFpEF in symptomatic outpatients, drawing on a combination of clinical and echocardiographic parameters—including body mass index, atrial fibrillation, age, and E/e′ ratio [60]. Conversely, the HFA-PEFF algorithm, endorsed by the European Society of Cardiology, provides a more comprehensive and multiparametric diagnostic framework that integrates functional, morphological, and biomarker domains. For patients with intermediate diagnostic probability, the use of stress-based modalities such as stress echocardiography or CPET is recommended to refine the diagnosis [26].

Efforts to further subclassify HFpEF into distinct phenotypes have yielded varying results, with current phenotype-based models demonstrating limited utility in clinical practice. Nonetheless, recent guidance from the European Society of Cardiology, including the position paper by Bonfioli et al., underscores that the presence of certain comorbidities can inform therapeutic direction [59]. Among these, cardiometabolic disorders (e.g., obesity, diabetes mellitus), atrial fibrillation, systemic hypertension, coronary artery disease, chronotropic incompetence, right ventricular dysfunction, and valvular pathology are particularly relevant.

The identification of coexisting or alternative pathologies that mimic or contribute to HFpEF is essential for accurate diagnosis and appropriate management. Conditions such as hypertrophic cardiomyopathy, cardiac amyloidosis (including both transthyretin [ATTR] and light-chain [AL] subtypes), Fabry disease, cardiac sarcoidosis, constrictive pericarditis, and pulmonary hypertension associated with WHO groups 1, 3, 4, and 5 necessitate distinct diagnostic pathways and therapeutic strategies [61,62].

Beyond its diagnostic precision, CPET offers profound prognostic value. The derived parameters, particularly a reduced peak VO_2_ and an elevated VE/VCO_2_ slope, have been consistently demonstrated to stratify HFpEF patients into distinct risk categories, offering predictive power for hospitalization and mortality that surpasses traditional resting echocardiographic or biomarker data alone, thereby enhancing the precision and efficacy of clinical care [63].

In conclusion, the effective diagnosis and management of HFpEF require a comprehensive and integrative strategy. Consequently, the systematic integration of CPET data into the clinical decision-making process is paramount. It provides an evidence-based foundation for tailoring therapeutic interventions—ranging from pharmacologic management to targeted exercise prescription and comorbidity optimization. By guiding these individualized management strategies, CPET holds the potential to not only refine patient care but also to improve functional capacity and, ultimately, long-term clinical outcomes in the heterogeneous HFpEF population.

## 9. Future Directions

Despite significant advances in understanding the complex pathophysiology of HFpEF, therapeutic options remain limited, and prognosis is still unsatisfactory for many patients. Recent breakthroughs, particularly with sodium–glucose cotransporter 2 (SGLT2) inhibitors, have shifted the therapeutic landscape. Large randomized controlled trials—EMPEROR-Preserved and DELIVER—have demonstrated that empagliflozin and dapagliflozin reduce the composite endpoint of cardiovascular death or heart failure hospitalization in patients with LVEF > 40%, with benefits largely driven by decreased hospitalizations and consistent across subgroups, including those without diabetes [64,65].

Mineralocorticoid receptor antagonists (MRAs) have also re-emerged as promising agents. The FINEARTS-HF trial reported that finerenone significantly reduced the risk of worsening heart failure events or cardiovascular death in HFpEF and HFmrEF, offering an alternative for patient intolerant to conventional MRAs [66].

Future therapeutic strategies are likely to focus on personalized medicine approaches, integrating phenotyping tools such as the H_2_FPEF score and HFA-PEFF algorithm to identify patient subgroups most likely to respond to specific interventions [26,60]. Moreover, targeting systemic inflammation, endothelial dysfunction, and skeletal muscle abnormalities—core pathophysiological drivers in HFpEF—may yield novel disease-modifying therapies [7,67].

Emerging modalities, such as inorganic nitrate supplementation, pulmonary vasodilators in select PH-HFpEF phenotypes, and structured exercise training programs, have shown early promise in improving functional capacity and quality of life [68,69]. As previously said, while the present data on HFpEF focus primarily on hard-endpoints and less on functional exercise testing, there is growing evidence from HFrEF populations showing that both dapagliflozin and vericiguat improved CPET parameters (peak VO_2_, anaerobic threshold, ventilatory efficiency) [70,71]. Thus, although data comes from HFrEF populations, it is clinically feasible that such therapies might favourably modify exercise physiology in HFpEF as well. This gap underscores the need for dedicated HFpEF trials incorporating CPET to determine whether functional improvements accompany the favourable clinical outcomes already emerging. Period CPET follow-up is crucial for maximizing the collection of objective functional capacity data and must be considered for reassessment with any patient deterioration or worsening of the NYHA class.

The integration of cardiopulmonary exercise testing (CPET) into therapeutic trials could facilitate more precise evaluation of interventions on central and peripheral determinants of exercise intolerance.

In patients with HFpEF, the exercise prescription also should be tailored to the individual’s CPET results. For instance, patients presenting with an elevated VE/VCO_2_ slope can benefit from structured, gradually intensifying supervised aerobic exercise, often combined with resistance programs, and potentially incorporating moderate-intensity interval training. The clinician should be focused on maximizing functional improvement tailoring exercise programs on CPET individual data.

Cardiopulmonary exercise testing (CPET) offers valuable insights that support precision medicine in HFpEF by enabling individualized phenotyping. By integrating key variables such as peak VO_2_, oxygen pulse, and VE/VCO_2_ slope, CPET can identify distinct patterns of exercise limitation and cardiovascular reserve. This personalized assessment helps tailor diagnostic evaluation, guide targeted therapeutic strategies, and monitor treatment response, moving beyond a one-size-fits-all approach and supporting a more patient-specific management of HFpEF.

Finally, the role of digital health tools—including remote monitoring of hemodynamic parameters and AI-driven risk prediction models—is rapidly expanding and could transform HFpEF management by enabling earlier detection of decompensation and more individualized follow-up strategies.

Overall, future research should prioritize multi-domain interventions targeting the heterogeneous mechanisms of HFpEF, validated in well-phenotyped patient populations, to bridge the current gap between mechanistic understanding and effective, durable therapies.

## 10. Conclusions

HFpEF remains a growing problem in daily clinical practice. CPET should be considered as an important part of clinical evaluation and may also support future therapeutic strategies. A better understanding of CPET parameters could help clinicians to recognize early functional impairment and to personalize treatment. Further research is needed to confirm these observations in larger populations.

## Figures and Tables

**Table 1:** CPET and HFpEF.

Study	Design	*n*	Inclusion Criteria/HFpEF Definition	Exclusion Criteria	CPET Parameters and Cut-Off Applied	Outcomes	Main Findings
Rozados da Conceicao et al., 2025 [30]	Prospective observational single center study	99	HFA-PEFF score NYHA ≥ II	Significant valvular disease, recent ACS, restrictive CM, CTx, COPD, severe CKD	PeakVO_2_ < 14 mL/min/kg VE/VCO_2_ slope > 34	Composite of HF hospitalization or CV death	VE/VCO_2_ slope significantly associated with outcome (HR 2.69, 95% CI 1.00–7.2) PeakVO_2_ not significantly associated with outcome (HR 1.34, 95% CI 0.60–2.9) On multivariate analysis, only VE/VCO_2_ slope independently associated with the outcome
Naito et al., 2024 [29]	Prospective observational study	240	HFA-PEFF score	Significant valvular disease, infiltrative, restrictive, or HCM, non-group 2 pulmonary arterial hypertension	PeakVO_2_ < 10 mL/min/kg VE/VCO_2_slope ≥ 45	Composite of HF hospitalization, all-cause death unplanned hospital visits requiring IV diuretics, or intensification of oral diuretics	Low peak VO_2_ in HFpEF was strongly associated with increased risk of adverse outcomes (HR 5.05, 95% CI 2.65–9.62) High VE/VCO_2_ slope in HFpEF conferred elevated risk compared to controls (HR 4.59, 95% CI 2.24–9.40) Abnormal CPET (peak VO_2_ < 10 mL/kg/min or VE/VCO_2_ slope ≥ 45) identified HFpEF patients at higher risk
Li et al., 2024 [32]	Prospective observational study	154	LVEF ≥ 50% with resting supine PCWP ≥ 15 mmHg and/or exercise PCWP/CO ≥ 2.0 mm Hg/L/min	AV node blocking drugs, AF, paced rhythm	%PredO_2_P < 85%	All-cause death	%PredO_2_P is an independent prognostic marker for all-cause death and those with higher %PredO_2_P exhibited longer survival
Gong et al., 2022 [33]	Retrospective observational single center study	585	Diagnosis of HF and LVEF ≥ 50%	Incomplete CPET data, LVAD or CTx	VE/VCO_2_ slope > 29	Composite outcome of all-cause death or HF hospitalization	VE/VCO_2_ slope was associated with increased risk of the composite outcome (HR 2.67, 95% CI 1.11–6.40)
Palau et al., 2018 [34]	Prospective observational study	74	Criteria for congestive HF and LVEF ≥ 50%	Recent ACS or AHF, significant lung disease, inability to perform CPET	ppMVO_2_	All-cause recurrent admission	A 10% decrease of ppMVO_2_ increased the risk of all-cause recurrent hospitalizations by 32% (IRR 1.32, 95% CI 1.03–1.68)
Nadruz Jr et al., 2017 [28]	Prospective observational study	195	Criteria for congestive HF and LVEF ≥ 50%	Missing baseline LVEF	PeakVO_2_ < 14.1 mL/min/kg VE/VCO_2_ slope > 30	Composite of all-cause death, LVAD or CTx, HF hospitalization	Peak VO_2_ and VE/VCO_2_ slope independently predicted HF hospitalization in HFpEF Prognostic impact of CPET variables per unit change was greater in HFpEF vs. HFrEF Adding CPET variables to clinical models improved risk prediction (C-statistic, NRI, IDI)
Sato et al., 2017 [19]	Prospective observational study	438	Criteria for congestive HF and LVEF > 50%	AHF or ACS, end stage CKD, end stage liver disease, advanced malignant disease	PeakVO_2_ < 14.1 mL/min/kg EOV	Adverse cardiac events (cardiac death and re-hospitalization for HF) and all-cause death	In HFpEF, peak VO_2_ was the main predictor of the outcome and mortality EOV was significant only in HFpEF, compared with HFrEF and HFmrEF
Klaassen et al., 2017 [35]	Retrospective observational study	88	Criteria for congestive HF and LVEF ≥ 45% and signs of pulmonary hypertension	Inability to perform CPET, significant valvular disease, significant lung disease	Peak VO_2_ VE/VCO_2_ slope	All-cause death Association of CPET parameters with hemodynamic variables (especially PVR)	Higher VE/VCO_2_ slope associated with worse NYHA class and higher NT-proBNP, independently predicted higher PVR Increased VE/VCO_2_ slope (but not peak VO_2_) was independently associated with all-cause death
Ali Shafiq et al., 2016 [27]	Retrospective observational study	173	Criteria for congestive HF and LVEF ≥ 50%	Previous aortic or mitral valve repair/replacement	ppMVO_2_ Peak VO_2_ VE/VCO_2_ slope EOV	Composite of all-cause death or CTx	ppMVO_2_ was the strongest predictor of the outcome, followed by peak VO_2_ VE/VCO_2_ slope and EOV had no significant association with the outcome
Yan et al., 2013 [36]	Prospective observational single center study	224	Criteria for congestive HF, LVEF ≥ 50% and LVDD on echocardiography	Recent ACS or ischemic stroke, dementia, severe lung disease, end stage CKD	Peak VO_2_ < 16.9 mL/min/kg VE/VCO_2_ slope ≥ 34.7	All-cause death or CV death	On multivariate analysis, VE/VCO_2_ slope, but not peakVO_2_, was independently associated with higher risk of CV death (HR 1.02, 95% CI 1.01–1.04) and all-cause death (HR 1.03, 95% CI 1.01–1.05)
Guazzi et al., 2008 [37]	Prospective observational multicenter study	556	Criteria for congestive HF, LVEF ≥ 50% and LVDD on echocardiography	Significant lung disease, or unable to perform maximal CPET	VE/VCO_2_ slope EOV	CV death	On multivariate analysis, VE/VCO_2_ slope and EOV, but not peakVO_2_, were independently associated with higher risk of CV death (HR 1.10, 95% CI 1.04–1.16 and HR 5.9, 95% CI 2.1–16.9)
Guazzi et al., 2005 [31]	Prospective observational study	409	Criteria for congestive HF and LVEF ≥ 40% (patients divided in DHF and SHF)	Significant obstructive lung disease	PeakVO_2_ < 14.1 mL/min/kg VE/VCO_2_ slope > 32	Composite of hospitalization or all-cause death	On multivariate analysis, similar association with outcome of VE/VCO_2_ slope and peakVO_2_ In DHF, VE/VCO_2_ slope better than peakVO_2_, remaining the only predictor regardless of LVEF

## Data Availability

No new data were created or analyzed in this study. Data sharing is not applicable to this article.

## Abbreviations

The following abbreviations are used in this manuscript:

ACS	Acute Coronary Syndrome
AHF	Acute Heart Failure
AF	Atrial Fibrillation
AV	Atrio-Ventricular
BNP	B-type Natriuretic Peptide
BMI	Body Mass Index
CA	Cardiac Amyloidosis
CKD	Chronic Kidney Disease
CM	Cardiomyopathy
CO	Cardiac Output
COPD	Chronic Obstructive Pulmonary Disease
CPET	Cardiopulmonary Exercise Testing
CTx	Cardiac Transplant
CV	Cardiovascular
C(a–v)O_2_	Arteriovenous Oxygen Content Difference
DHF	Diastolic Heart Failure
EOV	Exercise Oscillatory Ventilation
EDV	End-Diastolic Volume
eGFR	Estimated Glomerular Filtration Rate
E/e′	Early Diastolic Mitral Inflow Velocity to Mitral Annular Early Diastolic Velocity Ratio
HCM	Hypertrophic Cardiomyopathy
HF	Heart Failure
HFmrEF	Heart Failure with Mildly Reduced Ejection Fraction
HFpEF	Heart Failure with Preserved Ejection Fraction
HFrEF	Heart Failure with Reduced Ejection Fraction
HFA	Heart Failure Association
HFA-PEFF	Diagnostic algorithm for HFpEF
HR	Heart Rate
HTx	Heart Transplantation
IDI	Integrated Discrimination Improvement
IRR	Incidence Rate Ratio
IV	Intravenous
LV	Left Ventricle
LVAD	Left Ventricular Assist Device
LVDD	Left Ventricular Diastolic Dysfunction
LVEF	Left Ventricular Ejection Fraction
LVEDP	Left Ventricular End-Diastolic Pressure
MRA	Mineralocorticoid Receptor Antagonist
MRAs	Mineralocorticoid Receptor Antagonists
NO	Nitric Oxide
NTproBNP	N-terminal pro–B-type Natriuretic Peptide
NYHA	New York Heart Association
%PredO_2_P	Percent Predicted Oxygen Pulse
O_2_ pulse	Oxygen Pulse (Oxygen Uptake per Heart Rate)
PaCO_2_	Arterial Carbon Dioxide Tension
PCWP	Pulmonary Capillary Wedge Pressure
PetCO_2_	End-Tidal Carbon Dioxide Pressure
PH	Pulmonary Hypertension
ppMVO_2_	Percentage of Predicted Peak Oxygen Uptake
PVR	Pulmonary Vascular Resistance
ROS	Reactive Oxygen Species
SGLT2	Sodium–Glucose Cotransporter 2
SHF	Systolic Heart Failure
SV	Stroke Volume
SV_ACET	Stroke Volume by Acetylene Rebreathing
SV_ECHO	Stroke Volume by Echocardiography
TGF-β	Transforming Growth Factor Beta
TNF-α	Tumor Necrosis Factor Alpha
VD/VT	Physiological Dead Space to Tidal Volume Ratio
VE/VCO_2_	Ventilatory Equivalent for Carbon Dioxide Slope
VO_2_	Oxygen Consumption
VO_2_peak	Peak Oxygen Uptake
WHO	World Health Organization
